# Advantages of a combined rheumatoid arthritis magnetic resonance imaging score (RAMRIS) for hand and feet: does the RAMRIS of the hand alone underestimate disease activity and progression?

**DOI:** 10.1186/1471-2474-15-104

**Published:** 2014-03-26

**Authors:** Philipp Sewerin, Christian Buchbender, Stefan Vordenbäumen, Axel Scherer, Falk Miese, Ralph Brinks, Hans-Joerg Wittsack, Sabine Klein, Matthias Schneider, Gerald Antoch, Benedikt Ostendorf

**Affiliations:** 1 Department of Rheumatology, Univ Duesseldorf, Medical Faculty, Moorenstrasse 5, Duesseldorf D-40225, Germany; 2 Department of Diagnostic and Interventional Radiology, Univ Duesseldorf, Medical Faculty, Duesseldorf D-40225, Germany; 3 German Diabetes Center, Institute for Biometry and Epidemiology, Auf’m Hennekamp 65, Duesseldorf 40225, Germany

**Keywords:** Magnetic resonance imaging, Rheumatoid arthritis, RAMRIS, Therapy monitoring, Foot

## Abstract

**Background:**

To evaluate a combined rheumatoid arthritis magnetic resonance imaging score (RAMRIS) for hand and foot (HaF-score) in rheumatoid arthritis (RA).

**Methods:**

Magnetic resonance imaging (MRI, 0.2 Tesla) of the dominant hand and foot of 26 ACPA positive RA patients before and 6 months after initiation of methotrexate was obtained. RAMRIS of the hand was complemented by corresponding scoring of the foot (MTP I-V; HaF-score). Disease Activity Score 28 (DAS28) and a tender and swollen joint count (JC) of the joints scored in MRI were recorded. Changes in these scores (Δ) were assessed.

**Results:**

ΔHaF-score correlated significantly with ΔDAS28 (r = 0.820, 95%-CI 0.633-0.916). Correlations to ΔDAS28 were best for changes in the synovitis subscore (0.648) and bone marrow edema (0.703). Correlations to ΔDAS28 were significantly better for of the ΔHaF-score than ΔRAMRIS (0.499, 0.139-0.743, p = 0.0368).

All patients with at least moderate response (EULAR criteria, n = 11) had continuing disease activity on MRI, including five cases with new erosions, three of them at the feet. Improvements of the hand JC or foot JC were seen in 16 and 15 cases, respectively. However, MRI of the hand or feet improved in only 10 and 9 cases, respectively. No patient fulfilled SDAI remission criteria.

**Conclusions:**

The HaF-score identifies patients with continuing disease activity despite clinical response that would have been missed by consideration of the traditional RAMRIS or the DAS28 alone. Response as opposed to remission may be an insufficient goal in RA as all patients showed continuing disease activity, especially at the feet.

## Background

Rheumatoid Arthritis (RA) is a systemic inflammatory disease causing bone destruction and functional impairment predominantly of the small joints of hands and feet [1]. In order to impede destruction, remission became the utmost goal in the therapy of RA [2]. Beside effective treatment options including disease modifying anti-rheumatic drugs (DMARD) and biologicals, effective and sensitive tools for therapy monitoring are needed to reach this target. In daily practice as well as in research, therapy monitoring and response assessment are predominantly assessed by using the disease activity score 28 (DAS28) and correspondent response criteria as proposed by the American College of Rheumatology and the European League Against Rheumatism. The DAS28 comprises joints of the upper limbs, hands and the knees [3] but completely omits the feet. However, joint damage progression occur in patients considered to be in remission based on the DAS28, [4] especially in joints which are not covered by the DAS28. Consequently, there is evidence that the DAS28 might underestimate disease activity [5]. Nevertheless Smolen et *al.* demonstrated that the simplified disease activity index (SDAI), which is based on the same 28 joints used for calculation of the DAS28, has the highest predictive value for the development of new erosions [6].

In addition to clinical examination, imaging such as ultrasound and magnetic resonance imaging (MRI) play an important role in the management of RA patients. Although these high resolution imaging modalities are known to confer advantages as opposed to conventional radiographs of the hands and forefeet, the latter still state the gold standard for long-term evaluation of bone destruction. MRI, for instance, can depict earliest inflammatory joint changes such as bone marrow edema (BME), synovitis, or pre-erosion that are not visible on radiographs. Moreover, patients in clinical remission may display signs of disease activity in MRI and Power Doppler augmented ultrasound. These findings have a potential impact on therapeutic decisions, [7,8] because such findings were shown to provides a high predictive value for radiographic joint destruction and prognosis. Due to these data, MRI has become integrated part in the assessment of RA [9,10].

The RA- MRI- Scoring (RAMRIS) system, a standardized semiquantitative assessment of inflammatory soft tissue and destructive bone alteration, facilitated the use of MRI in outcome studies in RA [11-13]. Since most studies used the established RAMRIS method for the clinically dominant hand, little is known about inflammatory and radiomorphological changes of forefeet MRI regarding the relation to disease activity and response to DMARD therapy. Signs of joint inflammation of the foot on MRI were found to be as prevalent as in the hand [14]. They may even be present in the absence of inflammatory MRI findings of the clinically predominantly involved hand [15] as well as in the state of remission based on the DAS28 [16]. The latter study was the first to deploy the established RAMRIS system to the feet and has recently proofed to be highly reliable [17]. To our knowledge, there are no data available comparing the established RAMRIS score with a combined hand and foot score (HaF-score) for measurements of disease activity, radiological alteration and therapy response in RA.

The purpose of this study was to evaluate and compare the traditional RAMRIS of the hand with a new combined HaF-score in terms of clinical and serological correlation and sensitivity to change in RA patients before and after initiation of DMARD monotherapy of methotrexate (MTX), and to analyse the advantages of additional MR imaging of the foot.

## Methods

### Patients

Between November 2009 and July 2012, 26 consecutive patients were prospectively enrolled (18 female, 8 male; mean age 52.9, range ± 29.9 years, mean disease duration 8 weeks (SD 4.66, range 1–18 weeks), mean DAS28 3.5 (SD 0.78, mean CRP 0.9 mg/dl (SD 1.1)) All patients met 2010 American College of Rheumatology/European League Against Rheumatism Rheumatoid arthritis classification criteria [18] and were anticitrullinated peptide antibodies (ACPA) positive. 25 of 26 patients were positive for rheumatoid factor (RF). The general exclusion criteria for MRI imaging with gadolinium based contrast agent, were applied. Because of the dedicated open MRI system claustrophobia could be denied. Steroids were allowed up to a dose of 7.5 mg prednisolone at start and throughout the study. All patients received methotrexate at a dose of 15 mg, which could be taken orally or subcutaneously, conversions from oral to subcutaneous application were allowed. The dose of MTX was kept stable over 6 months. All patients were naïve to DMARD treatment before inclusion into the study and received MTX as part of standard care.

MRI of the dominant hand and foot was carried out prior to the initiation of MTX therapy and after 6 months. Routine parameters assessed during each visit included physical examination and routine laboratory tests including erythrocyte sedimentation rate (ESR) and C-reactive protein levels (CRP). This study was approved by the local review board and informed consent was obtained from all participating subjects.

### Magnetic resonance imaging

MR images of hands and feet were performed achieve most available comfort for patients in a low-field (0.2 T) dedicated open MR system (Esoate, C-Scan, Esaote Biomedica Germany GmbH). A dedicated wrist and ankle coil was used for image acquisition. The clinically dominant hand and foot (determined by an experienced rheumatologist) were imaged in a single session changing dedicated coil and patient position in every patient. MRI data was obtained on two consecutive time points (T0: prior to DMARD therapy, T1: after 6 month). The image protocol for the hand comprised the following sequences: Coronal Short tau inversion recovery (STIR) sequence with a field of view (FoV) of 180* 180 mm, matrix size 192* 152, slice thickness 3 mm (Time to repetition (TR) 2420 ms, echo time (TE) 26 ms, Time to inversion (TI) 85 ms), coronal 3 dimensional T1- weighted gradient echo sequence with a FoV of 180* 180* 60 mm, matrix size 192* 192* 40, slice thickness 1 mm (TR 50 ms, TE 16 ms) prior to and after intravenous injection of contrast material (0.2 ml/kg bodyweight of Gd-DTPA (Dotarem^©^, Guerbet GmbH, Germany)). The following protocol was used for imaging of the foot: Coronal STIR- sequence with a FoV of 190* 190 mm, Matrix size 192* 152, slice thickness 3 mm (TR 1700 ms, TE 22 ms, TI 80 ms), coronal 3 dimensional T1- weighted gradient echo sequence with a FoV of 180* 180* 70 mm, matrix size 192* 192* 40, slice thickness 1 mm (TR 50 ms, TE 16 ms) prior to and after intravenous injection of contrast material (0.2 ml/kg bodyweight of Gd-DTPA (Dotarem^©^)). The 3 dimensional T1-weighted gradient echo sequences of the hand and foot were additionally reconstructed in sagittal and axial planes. The overall image acquisition time was 39 minutes (18 minutes for the hand and 21 minutes for the foot). The plasma half-life time of Dotarem^©^ is about 90 min. Inter-reader reliability of MRI scoring was assessed by independent scoring of images at T0 by two different experienced radiologists (FM and CB) blinded to patient identity. The smalles detectable difference (SSD) according to Lassere is reported.

### Imaging data analysis

MR images were read in consensus by two board-certified radiologists with special expertise in musculoskeletal MRI and trained for RAMRIS scoring. Sites including for scoring on the hand MRI were: metacarpophalangeal joints II-V (MCP), carpal bones, distal radius, distal ulna, radiocarpal and distal radio-ulnar joint. At the foot the metatarsophalangeal (MTP) joints I-V were assessed. For each joint site, synovitis, BME and erosions were semiquantitatively graded as subscores according to the RAMRIS criteria [11]. The RAMRIS score of the hand was calculated. A combined hand and foot score (HaF-score) was calculated as a sum score of the RAMRIS and the MTP joint score including the subscores for synovitis, BME and erosions of each joint comparable to the calculation of the RAMRIS of the hand. The changes in the HaF-score or RAMRIS between the T0 and T1 (Δ) were further analyzed.

### Laboratory and clinical parameters

Laboratory and clinical parameters collected at baseline and follow-up were: ESR, CRP (mg/dl), DAS28 (based on CRP) and simplified disease activity index (SDAI). All clinical examinations were performed by an experienced rheumatologist. Changes of these parameters between T0 and T1 (Δ) were further analyzed.

### Ethic approval

The study was approved by the ethic committee of the medical faculty of the Heinrich-Heine University Duesseldorf (Study number 3226).

### Statistical analysis

Baseline and follow-up characteristics are described as proportions for categorical variables and as mean and standard deviation (SD) for continuous variables. Reported correlation coefficients are according to Spearman. Confidence intervals are two-sided 95% confidence intervals. Results with p <5% are considered to be significant. Confidence intervals for correlation coefficients have been calculated using the Fisher transform. Test for difference of two correlation coefficients has been accomplished as described previously [19]. Effect sizes are reported as standardized response means (SRMs) and are calculated according to Middle and van Sonderen [20]. Statistical analyses have been performed using R version 2.15.2 (R Development Core Team).

## Results

**Figure 1:**
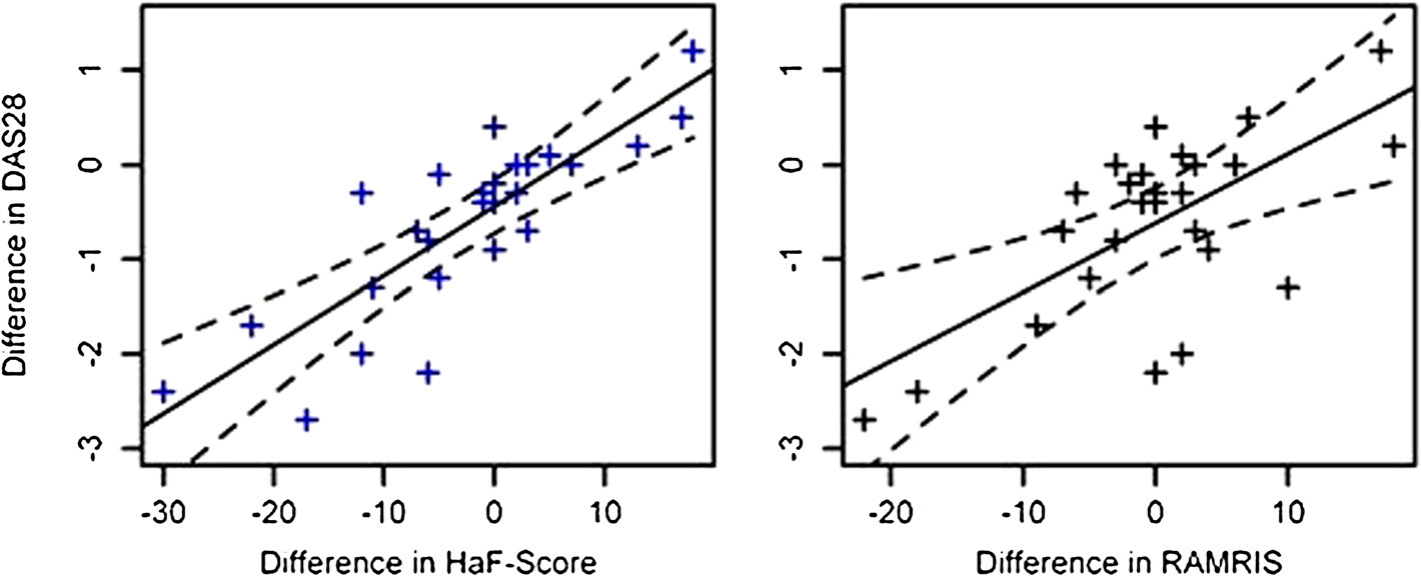
Scatterplot of differences: HaF-Score (left figure) and RAMRIS (right figure) vs. DAS28.

The mean value of DAS28 decreased from T0 (prior to MTX-therapy) to T1 (after 6 months) from 3.45 (min. 2.3; max. 4.9) to 2.9 (min. 1.8; max 4.6), the mean CRP decreased from 0.91 mg/dl (min. 0.3; max. 5.1) to 0.59 mg/dl (min. 0.3; max. 3.0). The mean RAMRIS decreased from 21.81 (min 0; max 53) to 21.69 (min 0; max 63) and the mean HaF-score from 33.58 (min 4; max 84) to 31.08 (min 2; max. 73) after 6 months (Table 1). ΔHaF-score showed the highest correlation with ΔDAS28 (T1-T0) (0.820 confidence interval (CI) 0.633-0.916) followed by Δsum score foot (0.522, CI 0.168-0.756) and ΔRAMRIS of the hand (0.662, CI 0.69-0.85) (Table 2, Figure 1). ΔHaF-score had a significantly higher correlation to ΔDAS28 than ΔRAMRIS (p = 0.0368). Correlations of ΔHaF-score to ΔSDAI values were overall slightly weaker (0.662 CI 0.369-0.835), with ΔSDAI demonstrating the highest correlations to ΔHaF-score amongst the parameters considered (Table 2). No patients reached remission based on SDAI criteria [3]. The evaluation of changes of the considered parameters (i.e. synovitis and BME) over time, employing the SRMs, showed a high effect size for the decrease of the DAS28 (-0.8188). In contrast, the effect size for the decrease in the HaF-score was trivial (-0.13). Similarly, based on the EULAR response criteria, eleven patients reached clinical improvement with good or moderate response. In the MRI follow-up (T1), all patients showed signs of continuing disease activity on MRI, including all good and moderate responder. Five of these showed actually new erosions (Table 3).

**Table 1:** Comparison of radiological, laboratory and clinical scores at baseline and after 6 month

**Score**		**T0 (baseline)**		**T1 (after 6 month)**		
	**Min.**	**Mean**	**Max.**	**Min.**	**Mean**	**Max.**
DAS28	2.3	3.45	4.90	1.8	2.90	4.60
SDAI	4.3	14.99	29.10	3.4	11.55	27.30
CRP	0.3	0.91	5.10	0.3	0.59	3.00
BSG	4.0	23.04	62.00	3.0	16.43	58.00
Sum score hand	0	7.59	22	0	6.385	25
Sum score wrist	0	14.27	42	0	15.31	59
Sum score foot	0	11.77	44	0	9.385	33
RAMRIS	0	21.81	53	0	21.69	63
HaF-score	4	33.58	84	2	31.08	73

**Table 2:** Spearman-correlation between ΔDAS28 and ΔSDAI to changes in other changes scores

**Change in score (**Δ**T1-T0)**	**Correlations between** Δ**DAS28 and changes in other scores**	**95% confidence interval**	**Correlations between** Δ**SDAI and changes in other scores**	**95% confidence interval**
**CRP**	0.170	-0.232–0.523	0.316	-0.082–0.626
**ESR**	0.217	-0.186–0.558	0.308	-0.09–0.621
**sum score hand**	0.368	0.022–0.661	0.280	-0.12–0.602
**sum score wrist**	0.449	0.075–0.713	0.335	-0.061–0.639
**sum score foot**	0.522	0.168–0.756	0.544	0.199–0.769
RAMRIS*	0.499	0.139–0.743	0.338	-0.057–0.641
HaF–score*	0.820	0.633–0.916	0.662	0.369–0.835

*Significant differences between ΔRAMRIS und ΔHaF-score (p = 0.0368) in correlation to ΔDAS28.

**Table 3:** Delta in the sum scores of the good or moderate EULAR-responders

**Sum score**	**Hand**		**Wrist**		**Foot**	
	**Mean**	**Range**	**Mean**	**Range**	**Mean**	**Range**
**Syn**	–2.545	–10–2	–2.545	–8–2	–3.455	–12–0
**Ero**	0.7273	–3–4	0.5455	–1–4	0.5455	–1–4
**BME**	–0.8182	–5–2	0.5455	–3–5	–3.273	–14–1

Syn = Synovitis, Ero = Erosion, BME = bone marrow edema.

The analysis of the different subscores of the HaF-score with ΔDAS28 revealed that changes in synovitis (0.648 CI 0.347-0.827) and BME (0.703 CI 0.434-0.857) were best correlated. In comparison to the combined HaF-score, MRI scoring for the foot alone, showed markedly lower correlations to ΔDAS28 (Δsynovitis 0.485 (CI 0.12-0.734) and ΔBME 0.514 (CI 0.159-0.752)) (Table 4).

**Table 4:** Spearman-Correlation between ΔDAS28 and synovitis-, erosion- and bone marrow edema-subscore between T0 and T1

**Score**	**Spearman-correlation**	**95% confidence interval**
**Syn overall**	0.648	0.347–0.827
**Ero overall**	0.125	-0.275–0.489
**BME overall**	0.703	0.434–0.857
**Syn hand**	0.487	0.123–0.736
**Ero hand**	-0.296	-0.613–0.104
**BME hand**	0.238	-0.164–0.573
**Syn wrist**	0.349	-0.044–0.649
**Ero wrist**	0.286	-0.114–0.606
**BME wrist**	0.326	-0.071–0.633
**Syn foot**	0.485	0.12–0.734
**Ero foot**	-0.282	-0.603–0.119
**BME foot**	0.514	0.159–0.752

Syn = Synovitis, Ero = Erosion, BME = bone marrow edema.

Next, the performance of the HaF-score against clinical examination was assessed. For this purpose, all joints considered in the HaF-score were examined on both sides and the number of swollen joints was added to the number of tender joints to create a sum score for the hand (hand joint count) and the foot (foot joint count). The hand count demonstrated worsening in six, unchanged values in four, and an improvement in sixteen patients. Despite improvement in the hand joint count on clinical examination, there was no improvement of the traditional RAMRIS in 6 of 16 patients. The foot joint count demonstrated worsening in two, unchanged values in nine, and an improvement in fifteen patients. Comparable to the traditional RAMRIS, the foot subscore of the new HaF-score uncovered 6 out of 15 patients with unchanged values or deterioration in spite of clinical improvement (i.e. foot joint count) (Table 5).

**Table 5:** Comparison between ΔRAMRIS of the hand (a) and the foot (b) and corresponding changes in total tender joint (TJ) and swollen joint (SJ) count

	**Differences in hand TJ-SJ count (T1-T0)**			
		**Improved**	**Equal**	**Worse**
	Improved	10	1	0
Δ**RAMRIS of the hand (T1-T0)**	Equal	2	2	3
	Worse	4	1	3
	**Differences in hand TJ-SJ count (T1-T0)**			
		**Improved**	**Equal**	**Worse**
	Improved	9	2	0
Δ**RAMRIS of the foot (T1-T0)**	Equal	2	6	0
	Worse	4	1	2

The pattern of inflammatory changes within the HaF-score was assessed. Overall, foot joints were more severely involved based on the MRI sum scores of synovitis, erosions and BME compared to the hand. Therein, MTP-2 was the single most affected joint. Furthermore, scoring of the MTP-2 showed the highest mean differences between T0 and T1 (Table 6).

**Table 6:** Most frequently affected joints

**Affected joint**	**Number of patients with Syn, Ero or BME at T0**	**Overall sumscore T0**	**Number of patients with Syn, Ero or BME at T02**	**Overall sumscore T1**	**Overall difference in the sum score**
**MCP2**	19	63	17	56	-7
**MCP3**	20	52	17	44	-8
**MCP4**	14	28	15	23	-5
**MCP5**	18	53	16	43	-10
**MTP1**	18	67	16	63	-4
**MTP2**	15	68	12	48	-20
**MTP3**	15	67	12	52	-15
**MTP4**	16	57	11	46	-11
**MTP5**	13	47	13	35	-12

MCP = metacarpophalangeal joints, MTP = metatarsophalangeal joints.

Finally, inter-reader reliability of MRI scoring at T0 was assessed to estimate the generalizability of HaF-scoring. SSD were as follows: RAMRIS: 4.77, HaF score: 4.60, RAMRIS subscore hand: 2.23, RAMRIS subscore wrist: 4.10, HaF subscore foot: 1.81. In 24 of 26 patients, the HaF subscore for the foot differed by only 1 or less. In addition we analysed the inter-rater agreement at T0 for the subscores Syn, Ero and BME for MCP-2 and MTP-2 as the most frequently involved joints. SDD were as follows for MCP-2: Ero subscore 0.91, Syn subscore 1.03. BME subscore 1.91; MTP-2: Ero subscore 0.87, Syn subscore 1.29, BME scubscore 0.87.

## Discussion

This study is the first to evaluate a combined hand and foot MRI score (HaF-score) for monitoring RA patients under DMARD therapy with methotrexate. A combined assessment on hand and feet is already established for years in the assessment of conventional radiographs [21-23]. However, MRI offers the potential for visualizing synovitis and BME, which was repeatedly shown to be predictive for radiologic progression [10]. Moreover, to our knowledge, this is the first systematic report of combined hand and foot MRI in one session in RA patients. Patients had to be summoned for examination and received contrast agents only once, resulting in a high patient satisfaction.

We found that the HaF-score correlated with clinical and laboratory measures of disease activity such as DAS28, SDAI and CRP. Analysing each component of the HaF-score separately, we found that changes in the HaF-score subscores for synovitis and BME, but not erosions, correlated with changes of the DAS28 and SDAI after therapy. This was also true if subscores for different anatomic regions were assessed separately (i.e. synovitis or BME for the hand, wrist and foot), albeit to a lesser degree. In comparison to clinical examination of the hand and the feet (hand and foot joint count), twelve patients with remarkable improvement in the joint counts after six months were uncovered by worsening or unchanged HaF-scores in MRI.

Numerous studies suggest that the DAS28 reflects disease activity [5,24]. A potential limitation to determining disease activity by the DAS28 allone is highlited by the finding of disease progression dispite clinical improvement or remission [25,26]. Krabben *et al.* have recently shown that subclinical inflammation - detected in hand and foot MRI - frequently occurs in ACPA positive patients [27]. Moreover, Wechalekar *et al.* demonstrated in a prospective study in RA-patients after six months of DMARD therapy DAS28 remisson rates of 30% (SDAI 28%), wherein 43% still showed active synovitis in the forefoot [28]. In accordance with this finding, we identified eleven RA patients as being in good or moderate response, based on the EULAR response criteria, who nevertheless showed signs of disease activity on MRI. Five of them had new erosions – and importantly, three of these erosions were located at the foot. Moreover there were seven patients with an improvement in (traditional hand) RAMRIS at T1 having new erosions, two of them in the foot. This is reflected by the effect size changes in the HaF-score, which were trivial while changes in DAS28 showed large effect sizes. Similarly, patients deemed to have improved based on the SDAI, a potentially more accurate clinical compound measure for the prediction of erosions in RA, [6] were uncovered to have continuing disease activity or deterioration based on MRI HaF-score. Importantly, in accordance with our finding that all patients had residual disease activity based on the HaF-score, no patient in the study reached remission based on SDAI criteria (SDAI < 3.3), which is currently considered to be the best predictive measure for radiological disease progression [3]. The same was true for all patients who reached remission in DAS28 while even all of them showed residual disease activity in the HaF-score. Hence, the current study stresses the importance of reaching remission rather than a moderate or good response only.

This study is limited by small patient number. Moreover, it could be argued that a clinical compound measure including the feet such as the DAS44 would have been more suitable for comparison with the new HaF-score. However, unlike the DAS44, the DAS28 is nowadays considered to be the gold standard for determining disease activity in RA, not only in studies, but also in clinical practice. Due to national guidelines for the application of X-rays, which allow routine conventional X-rays to be obtained only once a year, a comparison between changes in MRI and X-rays was not possible in the course of 6 months. Furthermore, additional MR parameters such as scoring of tenosynovitis might be of additional value, but were not featured in our study.

Based on the present results, longitudinal studies with a longer time period evaluating the potential of a combined hand and foot MRI score (HaF-score) to predict long-term radiological and functional outcomes are clearly warranted.

There is theoretical concern that generalizability of the HaF score may be hampered by difficulties in scoring the foot. However, inter-reader realiability for the HaF-score and especially the foot subscore was excellent in the present study. Thus, the HaF may be regarded as a reliable scoring system for the assessment of hand and foot inflammation.

## Conclusion

The HaF-score identifies patients with continuing disease activity despite clinical response that would have been missed by consideration of the traditional RAMRIS or the DAS28 alone. Response as opposed to remission may be an insufficient goal in RA as all patients showed continuing disease activity, especially at the feet.

## Abbreviations

ACPA: Anti-citrullinated protein antibodies; ACR: American College of Rheumatology; BME: Bone marrow edema; CI: Confidence interval; CRP: C-reactive protein; DAS28: Disease activity score 28; DMARD: Disease modifying anti rheumatic drug; EULAR: European league against rheumatism; ESR: Erythrocyte sedimentation rate; MRI: Magnetic resonance tomography; MCP joint: Metacarpophalangeal joint; MTP joint: Metatarsophalangeal joint; MTX: Methotrexate; RA: Rheumatoid arthritis; RAMRIS: Rheumatoid arthritis magnetic resonance imaging score; SD: Standard deviation; SDAI: Simplified disease activity index; SRM: Standardized response mean.

## Competing interests

The authors declare that they have no competing interests.

## Authors’ contributions

Study design: PS, CB, H-JW and BO. Study conduct: PS, CB, SV, SK, MS, GA and BO. Data collection: PS, CB, SV, AS, FM, SK and BO. Data analysis: PS, CB, SV, AS, FM, RB, SK and BO. Data interpretation: PS, SV, RB and BO. Drafting manuscript: PS. Revising manuscript content: PS, CB, SV, FM, RB, SK, MS, GA and BO. PS and RB take responsibility for the integrity of the data analysis. All authors read and approved the final manuscript.

## Pre-publication history

The pre-publication history for this paper can be accessed here:

http://www.biomedcentral.com/1471-2474/15/104/prepub
